# Development and feasibility of a theory-guided and evidence-based physical activity intervention in pregnant women with high risk for gestational diabetes mellitus: a pilot clinical trial

**DOI:** 10.1186/s12884-023-05995-7

**Published:** 2023-09-19

**Authors:** Xiao Yang, Zhixuan Xiang, Ji Zhang, Yingli Song, Erfeng Guo, Ruixing Zhang, Xin Chen, Lu Chen, Lingling Gao

**Affiliations:** 1 School of Nursing, Sun Yat-Sen University, No. 74 Zhongshan Road 2, Yuexiu District, Guangzhou, 510080 Guangdong Province P.R. China; 2 School of Nursing, Xiangtan Medicine & Health Vocational College, Xiangtan, China; 3 Zhengzhou Maternal and Child Health Care Hospital, Zhengzhou, China; 4 School of Nursing, Zhengzhou University, Zhengzhou, China

**Keywords:** Physical activity, Self-efficacy, Gestational diabetes, Prevention, Content validity, Feasibility

## Abstract

**Background:**

Physical activity has been utilized as an effective strategy to prevent gestational diabetes mellitus (GDM). However, most pregnant women with high risk for GDM did not achieve the recommended physical activity level. Furthermore, relevant physical activity protocols have varied without theory-guided and evidence-based tailored to pregnant women with high risk for GDM. This study aimed to develop and pilot test a theory-guided and evidence-based physical activity intervention protocol for pregnant women with high risk for GDM.

**Methods:**

The study design was guided by the Medical Research Council Framework for Developing and Evaluating Complex Intervention (the MRC framework). The preliminary protocol for physical activity intervention was developed based on self-efficacy theory, research evidence identified from systematic reviews and clinic trials, stakeholder engagement, context, and economic considerations. The preliminary intervention protocol was validated through a content validity study by an expert panel of 10 experts. A single-blinded randomized controlled trial (RCT) was designed to test the feasibility and acceptability of the intervention.

**Results:**

The validity of the preliminary intervention protocol was excellent as consensus was achieved. The final 13 sessions of self-efficacy enhancing physical activity intervention protocol were developed, including knowledge education, exercise clinic visits and video, and group discussions with face-to-face and online blended sessions. In the feasibility study, 34 pregnant women with high risk for GDM were randomized for the intervention (n = 17) or the control group (n = 17). The recruitment and retention rates were 82.9% and 58.9%, respectively. Women in the intervention group had a lower incidence of GDM (26.7% vs. 36.5%) than the control group (*P* >0.05). All participants were satisfied with the intervention and agreed that the intervention was helpful.

**Conclusions:**

The developed self-efficacy-enhancing physical activity intervention is a feasible and acceptable intervention for enhancing physical activity among pregnant women with high risk for GDM and is ready to be tested in a more extensive RCT study.

**Trial registration:**

The study was registered on 4 February 2022 (ChiCTR2200056355) by the Chinese Clini Trial Registry (CHiCTR).

**Supplementary Information:**

The online version contains supplementary material available at 10.1186/s12884-023-05995-7.

## Introduction

Diabetes is one of the most non-communicable diseases and the fastest-growing global health emergency in the 21st century. Gestational diabetes mellitus (GDM), defined as ‘glucose intolerance first detected during pregnancy’, is one of the risk factors for diabetes [1]. Women with a history of GDM appear to have a nearly 10-fold higher risk of developing type 2 diabetes (T2DM) than those with a normoglycaemic pregnancy [2]. Additionally, GDM is associated with long-term and short-term maternal and perinatal health outcomes [3].

The occurrence rate of GDM worldwide increased from 13.17% to 2020 to 16.7% in 2021 [4]. In mainland China, GDM is one of the most common complications of pregnancy with a prevalence of 14.8% [5]. Women with high-risk factors for GDM, such as obesity, older age, polycystic ovary syndrome, history of macrosomia, history of GDM, and family history of T2DM, were more likely to suffer GDM during pregnancy [6, 7]. The “three-child policy” is likely to predict a further increase in the incidence of GDM in mainland China [8], as the number of pregnant women with older age, pre-pregnancy overweight, or obese has risen dramatically since the “two-child policy” in mainland China [9]. There is a high priority to take strategies to prevent the occurrence of GDM focused on pregnant women with high risk for GDM in mainland China [10].

Physical activity can prevent and treat diabetes by improving glucose homeostasis and insulin sensitivity [11]. Evidence also indicates that physical activity during pregnancy could decrease the incidence of developing GDM [12, 13]. Guidelines worldwide recommend that women with healthy pregnancies achieve at least 150 min of physical activity at moderate intensity per week [14]. However, most pregnant women did not meet the recommended level of physical activity [15]. There were up to 97.2% of pregnant women under the recommended physical activity level goal in China [16]. Moreover, women with a high risk for GDM prefer to perform sedentary behaviors [17] and have low total physical activity levels [18].

Physical activity self-efficacy has been consistently identified as a key determinant for the beginning and maintenance of physical activity [19–21], as well as a mediator role in physical activity interventions [22]. Self-efficacy affects the selection of activities individuals choose to engage in, the degree of challenge they strive for when setting goals, and the amount of persistence and effort exuded in pursuing goals [23]. Higher self-efficacy is consistently associated with improved physical activity [24]. For pregnant women, physical activity self-efficacy has been identified as a modifiable theoretical factor associated with physical activity [25]. Intervention based on physical activity self-efficacy in women with GDM found improvement in physical activity self-efficacy, physical activity compliance, and effective blood glucose control [26].

It is suggested that standardized physical activity monitoring procedures must be conducted to increase physical activity and decrease the burden of non-communicable diseases [27]. Unfortunately, providing counseling on physical activity for pregnant women is not a routine service in the current antenatal care in mainland China and Western countries such as the United Kingdom [28]. In Chinese tradition, pregnant women obey traditional taboos such as ‘‘no jumping’’, ‘‘no moving heavy objects’’, ‘‘no fast walking’’, and ‘‘not too much walking’’ [29].

Mobile health (mHealth) technologies are cost-effective and scalable. mHealth programs use mobile and wireless technologies to support health and improve medical outcomes [30] and have been shown to produce modest improvements in several risk factors for non-communicable diseases [31]. Furthermore, mHealth interventions could foster small to moderate increases in physical activity, and the effects were maintained long-term [32]. However, adherence is a key challenge in any mHealth intervention program [33]. A national cluster-randomized controlled trial based on the mHealth program reported low engagement of intervention participants with the program, only approximately one-quarter of participants set a behavior change goal in the app or online [31]. It is suggested that digital health technologies might serve best as part of a larger overall health plan, supporting clinical practice and acting as healthcare companions, rather than working independently for patients trying to self-manage behavior change [34]. A recent systematic review indicated that face-to-face and mHealth blended interventions could lead to a significant increase in total physical activity levels among adults [35], as the strengths of one mode of delivery may compensate for the weaknesses of the other [36].

Pregnancy is a unique window in which pregnant women are easily motivated to maintain or start positive health behaviors for their own and the unborn child’s health, along with an increased frequency of contact with healthcare providers [37, 38]. All the above information highlighted a need for a theory-guided, evidence-based, face-to-face and mHealth blended physical activity intervention in women with high risk for GDM. This study was therefore conducted to develop, validate, and identify the feasibility and acceptability of a theory-guided, evidence-based, face-to-face and mHealth blended physical activity intervention in women with high risk for GDM in mainland China. The protocol of the self-efficacy-enhancing physical activity intervention in women with high-risk factors for GDM has been published elsewhere [39]. This study focused on the process of intervention protocol development and the feasibility and acceptability of the intervention protocol.

## Methods

### Overview of the research design

**Fig. 1:**
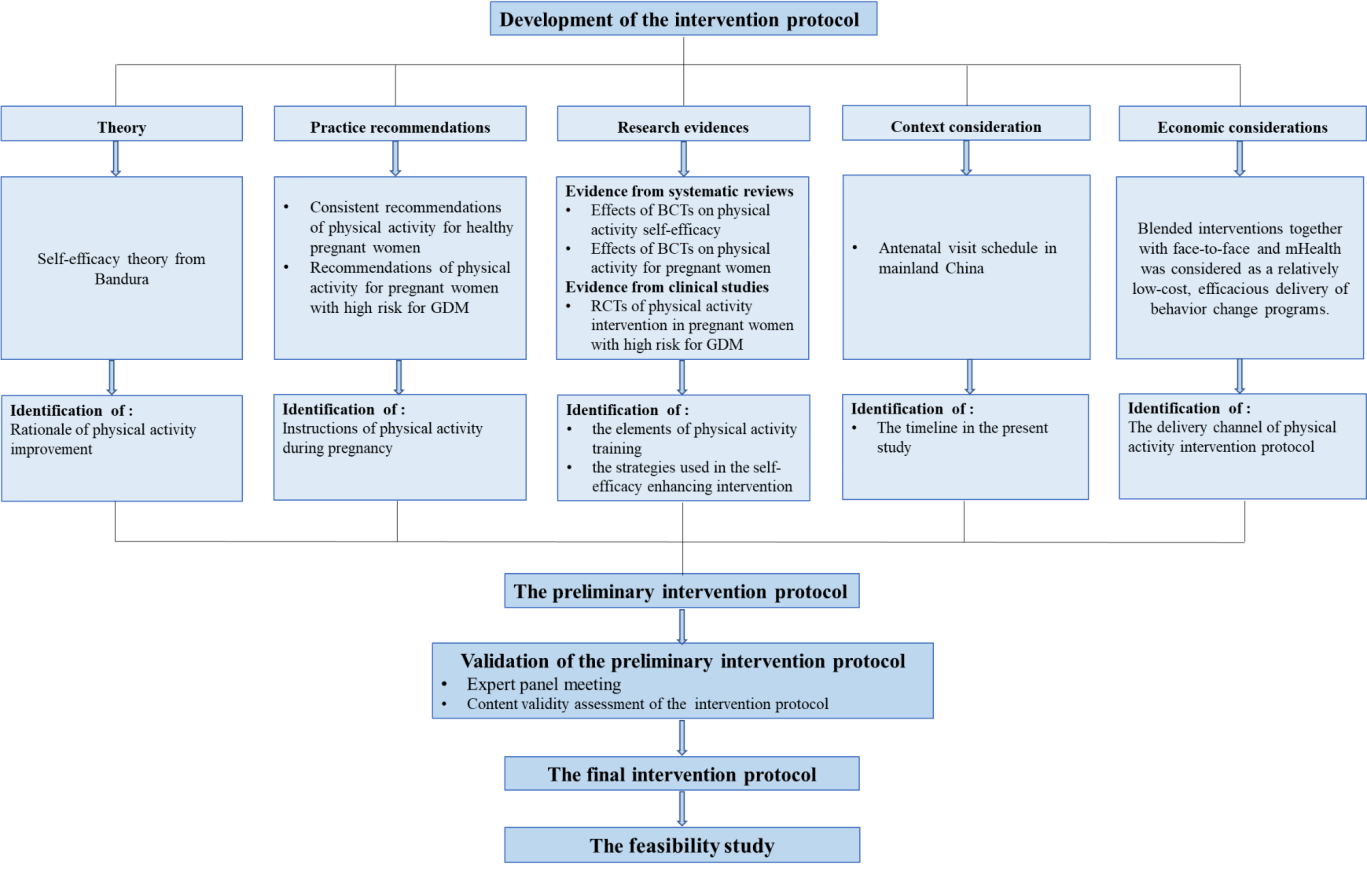
Overview of the research design Note: GDM, gestational diabetes mellitus; BCTs, behavior change techniques

The research design follows the Medical Research Council Framework for Developing and Evaluating Complex Interventions (the MRC framework) [40]. This study adopted phase I and phase II of the MRC framework to guide the development, validation, feasibility, and acceptability process of the evidence-based, theory-guided, face-to-face and mHealth blended physical activity intervention in women with high risk for GDM (Fig. 1), which contains: (a) development of intervention protocol; (b) validation of the intervention protocol; and (c) the feasibility study to examine the feasibility and acceptability through a single-blinded randomized controlled trial.

### Development of intervention protocol

#### Identification of the physical activity theories

Bandura’s self-efficacy theory was identified by reviewing a theoretical framework for physical activity intervention [41] to guide the development of the intervention in this study.

Self-efficacy theory is one of the most prominent psychological theories about behavior change and lays its foundations on self-efficacy [42, 43]. Moreover, physical activity self-efficacy has been identified as a modifiable theoretical factor associated with physical activity during pregnancy [25]. Self-efficacy develops due to four sources of information: past performance accomplishments, vicarious experiences, verbal persuasion, and physiological and emotional status [43]. Among them, past performance accomplishments are the most powerful source of self-efficacy [42, 44]. It refers to the direct experience of performing a specific task and, hence, it represents an authentic indicator of the individual ability to accomplish similar tasks in the future. Experiences interpreted as successful generally increase confidence while unsuccessful generally undermine it. The behavioral goal should be set gradually to obtain the achievement of successful experiences. A vicarious experience could be defined as a person’s reinforcement of self-belief by watching a similar individual succeed in certain situations (i.e., “if they can do it, I can do it”). Verbal persuasion refers to guiding the individual to believe that they can succeed in specific situations through positive feedback and verbal clues (i.e., “Good job. You are so great”). Physiological and emotional status refers to how an individual’s physiological state and their interpretation of that state can affect whether an experience is empowering or disempowering for them.

#### Identification of practice recommendations

The research team conducted a systematic review to identify consensus recommendations for physical activity for pregnant women [14]. The systematic review indicated that women without medical contraindications should be physically active throughout the pregnancy. All healthy pregnant women should achieve moderate physical activity for at least 150 min per week, to be physically active 30 min per session on at least five days, and preferably all days of the week. Aerobic and resistance training activities are highly recommended with gradual warm-ups and cool-downs. Moreover, the guideline recommended that pregnant women at high risk for GDM start to do physical activity in the early second trimester if previously sedentary [45]. Subjective assessment of moderate intensity was suggested as individuality and convenience. The intensity of physical activity was controlled by the Borg Scale [46] and Talk Test [47]. The Borg’s scale is called the Borg Rating of Perceived Exertion Scale. It has often been used to monitor and quantify an individual’s perceptions of effort during exercise as the perceived effort correlates relatively well with heart rate. The scale score ranges from 6 to 20, indicating that exercise is perceived as “no exertion at all” to “very hard.” Scores 6 to 11 and 12 to 14 were defined as very or fairly light in light intensity and somewhat hard in moderate intensity. Talk Test is a valid, reliable, practical, and inexpensive tool for prescribing and monitoring exercise intensity. The light intensity is when one can talk and sing during exercise. Moderate intensity is when one can talk but not sing during exercise.

#### Identification of evidence base

The literature search was conducted through 11 English and Chinese electronic databases, including Medline, PubMed, Web of Science, Cochrane Central Register of Controlled Trials, Cochrane Database of Systematic Review, Scopus, Embase, PsycINFO, China National Knowledge Infrastructure (CNKI), Wan Fang Data and Chinese Medical journal database. The articles were searched from the earliest dates to February 2023. The search terms used were “gestational diabetes”, “exercise”, “physical activity”, and “risk”. Randomized trials that reported on physical activity relevant to gestational diabetes prevention and were published in English or Chinese were considered eligible. The following were excluded: (a) conference abstracts, case reports, and review papers; (b) duplicated articles; (c) articles lacking original data or with inaccessible data; and (d) articles for which the full article was unavailable. A total of 1956 articles were searched, and 6 articles were identified through a reference list. Duplicate articles (n = 760) were removed. The preliminary intervention protocol framework was developed by 12 randomized controlled trials (RCT) focused on physical activity intervention in pregnant women with high risk for GDM [48–59]. The enrollment diagram is shown in Figure S1. Two reviewers (XY and XC) independently assessed the risk of bias in eligible studies by using the Cochrane tool for bias risk assessment [60]. A meeting with the third reviewing author (ZXX) was arranged when disagreement persisted after discussion between the two reviewers. Table S1 presents a summary of the risk of bias in the included studies.

#### Consideration of the context’s characteristics

In mainland China, pregnant women are suggested to be seen every 4 weeks until 28 gestational weeks, every 2 weeks until 36^+ 6^ gestational weeks, and then weekly until giving birth. It is estimated that a woman “booking” at 6–12^+ 6^ weeks’ gestation and delivering at 40 weeks makes 11 antenatal care visits [61]. In mainland China, pregnant women usually take an oral glucose tolerance test at 24–28 gestational weeks to diagnose GDM [61]. Thus women will be recruited at the time of booking into the hospital at < 12^+ 6^ weeks’ gestation. The Follow-up will occur at 24–28 weeks, 35–37 weeks, and within 3 days after delivery.

#### Economic considerations

Blended interventions together with face-to-face and mHealth were considered as a relatively low-cost, efficacious delivery of behavior change programs. During early pregnancy, the usual prenatal care visits are infrequent, so it’s a good time to add mHealth sessions between the prenatal care visits.

WeChat is one free application of the most popular social media platforms released in 2011 by Tencent. WeChat offers sharing moments, mobile payment, small programs, public accounts, free phone calls, and instant messaging with text, pictures, or videos, not only person-to-person but also group discussions. With its convenience and various services, WeChat has become the most prevalent social networking platform in China [62]. Thus, we applied WeChat as the mHealth method for uploading physical activity video and daily communication. Tencent meeting is another free application to hold meetings with groups. Tencent meeting offers speech, discussion, and chatting with the camera on or off. Tencent meetings will be used as a delivery channel every two weeks for group discussions in online sessions in the present study.

### Validation of the intervention protocol

The expert panel discussion was applied to achieve experts’ opinions and validate the intervention protocol via a content validity study. Inclusion criteria for the expert were: (a) any gender; (b) specialized in obstetrics, nursing, sports, physiotherapy, psychology, or research; (c) 10 years or more experience in their specialized fields; (d) Bachelor’s degree or higher; (e) voluntarily participate in the consultation. The experts discussed the readability and rationale of the preliminary intervention protocol through the online discussion. The discussion continued until the experts reached an agreement. The research team then considered the experts’ suggestions for improving and refining the preliminary intervention protocol. The discussion was documented through audio and notes.

The content validity assessment form consists of 4 primary indicators, 10 secondary indicators, and 17 tertiary indicators addressed from the preliminary intervention protocol. Experts were asked to rank the importance of each item on a Likert 5-point scale, ranging from 1 (not at all important) to 5 (very important) [63]. An expert questionnaire was collected, including demographic characteristics, familiarity coefficient (Cs), and judgment coefficient (Ca). Demographic characteristics of the experts included gender, age, profession, institution, academic professional rank, highest academic qualification, and years of professional experience. The Cs is the familiarity with the content of protocol from the experts’ view [64]. The Ca is the judging criteria for the comments with four categories [64].

### Feasibility study

#### Design

A single-blinded randomized controlled trial was performed for the examination of the feasibility and acceptability of the self-efficacy-enhancing physical activity intervention. Eligible participants were recruited and randomly allocated to receive physical activity intervention or usual care.

#### Sample

The participants were recruited from the obstetric clinic of the study hospital. Eligible pregnant women were Zhengzhou citizens who were 18 years old or above, singleton pregnancy, less than 12^+ 6^ weeks pregnancy, have at least one risk factor for GDM (e.g., maternal age ≥ 35 years old, pre-pregnancy BMI ≥ 24 kg/m^2^, family history of type 2 diabetes, polycystic ovary syndrome, previous macrosomia, previous GDM, previous glucose intolerance, previous fetal anomaly or hydration, current pregnancy with fetus growing than the gestational age, hydration, or repeated colitis) [65, 66]. Women were excluded if they: (1) had an exercise contraindication [14]; (2) were participating in other antenatal physical activity programs; (3) or were currently being treated with metformin or corticosteroids.

The previous study recommended 12 participants per group for pilot studies [67]. A similar sample size (29 participants) is seen in comparable pilot studies conducting physical activity interventions for general pregnant women [68]. In the present study, we aimed to recruit at least 30 pregnant women at high risk for GDM.

#### Intervention

The participants in the intervention group received the developed self-efficacy-enhancing physical activity program. Both intervention and control group participants received usual antenatal care, including routine antenatal visits and free antenatal pregnancy school.

#### Outcomes

According to the MRC framework, the feasibility stage includes testing procedures for acceptability and estimating the recruitment and response rates. Therefore, this study estimated the intervention program from the following aspects: (1) rates of recruitment, (2) retention rate in the study, (3) success rate of sessions participation, (4) acceptability of the intervention: A 8-item satisfaction questionnaire was developed to measure participants’ satisfaction with the intervention. Participants in the intervention group were asked to rank the satisfaction of each item on a Likert 5-point scale, ranging from 1 (not at all agree) to 5 (strongly agree). The satisfaction rate was calculated using the percentage of participants who rated an item as 4 or 5 points. The Cronbach’s α of the 8-item satisfaction questionnaire was 0.914 in the present study.

### Data analysis

SPSS 26.0 was used to analyze the quantitative data. Descriptive statistics such as mean, standard deviation coefficient of variation (CV), and consensus level of agreement (CLA) were calculated. Two-sample independent *t*-tests or χ^2^ test were used to compare demographic characteristics between participants in the intervention and control group. The consensus threshold was defined as the mean (≥ 4.0), CV (< 0.25), and CLA (≥ 70%) [69]. The CLA was calculated using the percentage of experts who rated an item as 4 or 5 points. The reliability and representation of the expert consultation were measured according to the positive feedback rate, authority coefficient (Cr), and Kendall’s coefficient of concordance (Kendall’s W). The positive feedback rate was the rate of return of expert questionnaires. The Cr was calculated by using the formula of Cr = (Cs + Ca)/2 [70]. The levels of Cs and Ca were valued according to the previous study [71]. A Cr of above 0.7 is considered to be reliable, and a Cr of 0.8 indicates even higher reliability of the expert’s judgment [71, 72]. Kendall’s W (ranging from 0.0 to 1.0) was used to examine the level of expert interrater agreement [73]. The closer the value to 1.0 indicated the greater the positive correlation [74].

### Ethical consideration

The study was approved by the institutional review board of the university and study hospital (No. L2022SYSU-HL-004). The experts were fully informed of the purpose, significance, research contents, and methods of the study. Informed consent was obtained from all experts included in the study. All the participants in the pilot clinical trial voluntarily signed the informed consent, with the assurance of the confidentiality of personal information and the security of data. The participants were informed that there was no additional fee to participate in the study. They could withdraw from the study at any time.

## Results

The results of this study include components of (1) the preliminary intervention protocol; (4) validation of the preliminary intervention protocol; (5) the final intervention protocol; and (6) the feasibility and acceptability of the intervention protocol.

### The preliminary intervention protocol

#### Identification of the elements of physical activity training

The elements of physical activity training were developed based on evidence adopted from a systematic review [14], clinical practice recommendations [45], and 12 RCTs [48–59]. All the participants aimed to achieve a 30-minute structured physical activity program in moderate intensity on at least 5 days per week from 13–14^+ 6^ gestational weeks to 35–37 gestational weeks. The moderate intensity of the physical activity will be set according to each woman’s perceived effort (within the range of 12–14 points on the Borg Scale) [46] and Talk Test (one can talk but not sing during exercise) [47].

The physical activity session lasts for 40 min, including a 5-minute warm-up, a 30-minute main exercise section, and a 5-minute cool-down. The movements in the physical activity training were validated by the sports coach and the previous RCTs. The session started and ended with light-intensity, 5-minute activities that consisted of walking, breathing training, and static stretching of most muscle groups, including upper limbs, lower limbs, neck, back, and trunk muscles.

The main part of the physical activity session lasted 30 min and included moderate-intensity aerobic exercises and resistance training. Aerobic exercise consists of brisk walking, involving the upper and lower limbs, and stretching activity. The resistant training engaged major muscle groups, including pectoral, back, shoulder, and upper and lower limb muscles. The movements consisted of half-squats by own body weight, arm extensions, arm side lifts, arm elevations, shoulder shrugs and rotations, lateral leg elevations, knee extensions, and knee (hamstring) curls. Exercises that involved extreme stretching and joint overextension, ballistic movements, or jumps were avoided. All the movements were done in the standing position to avoid the supine position on the floor. Aerobic and resistance exercises will be repeated 10–15 times for each movement. Participants can adjust the exercise intensity and frequency according to the progress of their pregnancy. The physical activity training was recorded in video with modeling by a gymnastics student. The core elements of the physical activity training for pregnant women with high risk for GDM are shown in Table 1.

**Table 1:** Core elements of physical activity training for pregnant women with high risk for GDM

	Elements	Content	Major movements	Evidence (references)
Warm up		5-minute started at light intensity before the main session	walking, breathing training, and static stretching of most muscle groups, including upper limbs, lower limbs, neck, back, and trunk muscles.	[14, 50–52]
main session (FITT)	Frequency (F)	at least 5 days, prefer everyday per week	Aerobic exercise consisted of brisk walking, involving the upper and lower limbs, and stretching activity. The resistant training engaged major muscle groups, including pectoral, back, shoulder, and upper and lower limb muscles. The movements consisted of half-squats by own body weight, arm extensions, arm side lifts, arm elevations, shoulder shrugs and rotations, lateral leg elevations, knee extensions, knee (hamstring) curls. Each movement will be repeated 10–15 times.	[14, 45, 48, 49, 53–57]
	Intensity (I)	moderate-intensity assessed by Borg Scale and Talk Test		
	Time (T)	30 min per session		
	Type (T)	Aerobic activities and resistance training		
Cool down		5-minute performed at light intensity after the main session	the same exercises as the warm-up period	[55]
Total duration		from 13–14^+ 6^ gestational weeks to 35–37 gestational weeks.		[45, 55, 58]
Follow-up visit		first visit: 24–28; second visit: 35–37; third visit: 72 h within delivery		[48, 59]
Compliance		exercise diary; a log of exercise activities and attendance		[54, 56]

#### Identification of the strategies used in the self-efficacy enhancing intervention

According to Bandura [43], self-efficacy is constructed from four main sources: performance accomplishments, vicarious experiences, verbal persuasion, and physiological and emotional status. A recent review revealed that behavior change techniques (BCTs) were significantly and positively correlated with post-intervention changes and maintained changes in physical activity self-efficacy [75]. A BCT refers to an active ingredient of an intervention that aims to change an individual’s normal behavior. It can be observed, replicated, and irreducible [76]. BCTs can be adopted alone or in combination, with more BCTs may be more effective for maintaining changes in physical activity self-efficacy [75]. Thus we applied BCTs together with four main sources of self-efficacy to enhance physical activity self-efficacy in pregnant women with high risk for GDM.

The strategies of the physical activity self-efficacy enhancing intervention were supported by the evidence of self-efficacy theory [42, 43], five systematic reviews of BCTs associated with changes in physical activity self-efficacy [75, 77–80], and a review of BCTs associated with physical activity promotion in pregnant women [81]. BCTs with a positive relationship with physical activity self-efficacy were extracted. Table 2 outlines BCTs and examples of the four strategies.

**Table 2:** Strategies used in the self-efficacy-enhancing intervention

	Strategies	BCTs	Evidence (references)
Performance accomplishments	Identifying the obstacles to keeping participants active through discussion	Problem solving	[42, 43, 81]
	Setting achievable goals and actions, e.g., achieving 10 min of exercise following the exercise video daily before increasing gradually to 30 min	Goal setting; Set graded tasks;	[42, 43, 80, 81]
	Negotiating techniques with participants to achieve bigger goals, e.g., set alarm on phone for activity; put notes on doors, the refrigerator, or the television to be active; stand or walk rather than siting in add breaks	Prompt self-monitoring of behaviour	[42, 43, 79–81]
	Monitoring physical activity diary and gestational weight gain on WeChat notes	Prompt self-monitoring of behaviour	[42, 43, 79–81]
	Planning for decreasing sedentary behavior	Prompt self-monitoring of behaviour	[42, 43, 79–81]
	Providing positive feedback for participants’ accomplishments	Provide feedback on performance	[42, 43, 77, 78, 81]
	Providing booklet to reinforce knowledge	Provide instruction	[42, 43, 78, 81]
	exercise clinic visit	Behavioral practice/rehearsal, Demonstration of the behavior	[75, 81]
Vicarious experience	Checking behavioral tracking, review, and feedback on goals; “we’re going to check how you went with your physical activity and tracking and work together to set a healthy activity goal.”	Prompt review of behavioural goals	[42, 43, 79]
	Sharing self-management strategies from successful pregnant women	Facilitate social comparison; social support	[42, 43, 78, 81]
Verbal persuasion	Discussing and providing information about consequences of physical inactivity and unhealthy gestational weight gain	Provide information on consequences of behaviour	[42, 43]
	Confirming participants have the capability for exercise and weight self-management	Motivational interviewing	[80]
	Informing that one’s own behavior may be an example to others, i.e., inform the participants that if they do physical activity, that may be a good example for their friends and family members.	Information about social and environmental consequences	[75]
	Guiding participants to recall previous successful behavior-change situations, discuss context and factors associated with success	Prompting focus on past success	[80]
	Providing positive feedback for the participant’s effort	Reinforcing effort or progress towards behaviour;	[78]
physiological and emotional	Assessing and explaining the participant’s pregnancy-related symptoms and negative emotions	Stress Management/emotional control training	[42, 43, 75, 79, 80]
	Discussing strategies for managing symptoms, anxiety, or depression, such as positive self-talk and muscle relaxation		

Note: BCTs, Behavior Change Techniques

#### Identification components of the preliminary intervention protocol

Based on the self-efficacy theory and evidence above, the preliminary component of the intervention protocol was addressed. Table 3 shows detailed information on the preliminary intervention protocol. The intervention will start from 13–14^+ 6^ gestational weeks till 35–37 gestational weeks. The total duration of the intervention will last 24 weeks. A previous study showed that pregnant women preferred to meet other pregnant women in a similar situation to reach peer support in antenatal classes [82]. In addition, the perception of others’ achievement in the targeted activity was better to help enhance physical activity self-efficacy during pregnancy [83]. Therefore, the intervention will be delivered in a small group of 15–20 pregnant women who are in a similar expected date of confinement (EDC).

**Table 3:** The preliminary intervention protocol

Primary indicators(theory components)	Secondary indicators	Tertiary indicators	Time	Delivery type	Duration	Intervener
1. Accomplishment experiences	1.1 knowledge education session	1.1.1 What is GDM? 1.1.2 Adverse health outcomes of GDM? 1.1.3 Physical activity could prevent GDM. 1.1.4 The frequence, intensity,type, time of physical activity during pregnancy	13–14^+ 6^ weeks’ gestation	face to face in groups with 15–20 participants	about 40 min	Researcher, nurse and midwife
	1.2 exercise clinic visit	1.2.1 the coach teach pregnant women how to exercise safely 1.2.2 the coach guide the participants to do physical activity together following the exercise video. 1.2.3 uploaded the exercise video to WeChat platform 1.2.4 teach the participants how to keep exercise diary	13–14^+ 6^ weeks’ gestation	face to face in groups with 15–20 participants	about 50 min	Researcher, coach, obstetricians, nurses, and midwife
2. Vicarious experience	2.1 positive feedback	2.1.1 Checking behavioral tracking, review, and feedback on goals	every two weeks from 15–16^+ 6^ to 37 weeks	online group discussion with 15–20 participants via Tencent Meeting	about 15–20 min	Researcher, nurse and midwife
	2.2 role model	2.2.1 Sharing self-management strategies from successful pregnant women				
3.Verbal persuasion	3.1 reminder	3.1.1 Daily reminders via the WeChat group to encourage the women to follow the 40-min video, performing the exercises and recording in their exercise diary	every two weeks from 15–16^+ 6^ to 37 weeks	online group discussion with 15–20 participants via Tencent Meeting	about 15–20 min	Researcher, nurse and midwife
	3.2 problem solving	3.2.1 Discuss the problems that arise when doing exercise and share solutions on how to keep active with each other				
	3.3 encourage	3.3.1 Confrming participants have the capability for exercise and weight self-management				
	3.4 recall previous successful experience	3.4.1 Guiding participants to recall previous successful behavior-change situations, discuss context and factors associated with success				
	3.5 positive feedback	3.5.1 Providing positive feedback for the participant’s effort				
4. physiological and emotional status	4.1 knowledge education session	4.1.1 Assessing and explaining the participant’s pregnancy-related symptoms, and discussing strategies for managing symptoms, such as muscle relaxation	15–16^+ 6^ weeks’ gestation	online group discussion with 15–20 participants via Tencent Meeting	about 30 min	Researcher, nurse, and midwife
		4.1.2 Assessing and explaining the participant’s negative emotions, and discussing strategies for managing anxiety, or depression, such as positive self-talk	17–18^+ 6^ weeks’ gestation	online group discussion with 15–20 participants via Tencent Meeting	about 30 min	Researcher, nurse, midwife, and psychological consultant

Note: GDM, gestational diabetes mellitus

The face-to-face knowledge education session and exercise clinic visit will be conducted to help pregnant women achieve accomplishment experiences. The knowledge education session aims to emphasize the positive influence of physical activity during pregnancy on GDM prevention. The clinical practice recommendations regarding physical activity during pregnancy were included. After the knowledge education session, the coach will conduct a face-to-face group exercise clinic visit to teach pregnant women how to exercise safely. Then the coach guides the participants to do physical activity together following the exercise video under the supervision of obstetricians, nurses, and midwives. The exercise video will be uploaded to the WeChat platform. Then the researcher will teach the participants how to keep an exercise diary. The participants will be suggested to begin by completing 10 min of moderate physical activity daily and increasing the duration gradually to 30 min.

Vicarious experience and verbal persuasion will be achieved through online group discussion via Tencent Meeting. Previous research indicated that two weeks of inactivity was one of the most significant predictors of dropouts among health platforms for behavioral intervention [33]. Therefore the online group discussion in the present study will be conducted every two weeks from 15–16^+ 6^ to 37 gestational weeks. Several strategies will be adopted to enhance the physical activity self-efficacy of pregnant women, including positive feedback on previous physical activity goals and the participant’s effort, role models from successful pregnant women, encouragement, and reinforcement of the participants’ capability and previous successes, discussing the problems that arise when doing exercise and share solutions on how to keep active with each other, and daily reminders via the WeChat group to encourage the women to perform exercises following the exercise video and record the exercise diary.

The physiological and emotional status will be reached by knowledge education sessions through online group discussions via Tencent Meeting. During 15–16^+ 6^ gestational weeks, knowledge education about pregnancy-related symptom management will be provided by the researcher, obstetric nurses, and midwife. During 17–18^+ 6^ gestational weeks, knowledge education about pregnancy-related emotion management will be offered by researchers, obstetric nurses, midwives, and psychological consultants.

### Validation of the preliminary intervention protocol

Ten experts participated in the discussion session. The expert panel consisted of an obstetrician, a physiotherapist, 2 coaches, a psychologist, 4 clinical nurses, and a midwife. The characteristics of the expert panel members are presented in Table S2. Ten questionnaires were distributed and ten effective questionnaires were returned, with a recovery rate of 100%, indicating a higher enthusiasm of the experts. The Cs were 0.84 (SD 0.13), Ca was 0.92 (SD 0.07) and Cr was 0.88 (SD 0.09), which meet the standard of expert consultation authority coefficient > 0.7 (Table S3). The Kendall’s W of the preliminary intervention protocol was 0.321 (χ^2^ = 96.163, *p*<0.001), which indicated a greater positive correlation (Table S4). The content validation index of all items met the acceptable consensus level (Mean, 4.20-5.00; CV, 0.00-0.19; CLV, 80.00-100.00%) (Table S5).

The experts claimed that the number of 15–20 participants in a small group may make it difficult for participants to deeply communicate with each other. According to their clinical experience, they suggested that 8–10 participants in a small group were more effective. The experts think that more online sessions may lead to a higher rate of dropouts. It’s better to add more face-to-face sessions. As the pregnant women in small groups with similar EDC, the face-to-face session could be added according to the antenatal visits following the pregnancy progress.

In addition, the experts claimed that some pregnant women refuse to engage in physical activity for safety precautions due to the Chinese traditional taboos. It is necessary to know the safety precautions when doing physical activity during pregnancy. Pregnant women will be more motivated to begin and maintain physical activity if they feel safe when doing physical activity. In addition, women will have physiological weight gain following the pregnancy’s progress. Weight gain is a visual indicator for pregnant women. Regular physical activity during pregnancy could help to control weight gain within a reasonable context. Pregnant women will be more confident to keep moving when perceiving the positive effect. Knowledge regarding healthy weight gain during pregnancy is needed in the education session. Except for the exercise diary, every day’s weight is suggested to be recorded in the diary too.

Experts claimed that group discussion was one of the most effective approaches to enhancing physical activity self-efficacy in the present study. Pregnant women may shape better confidence by having more chances to speak out and share their experiences with others. The role of the intervener in the group discussion is as a toastmaster to ask questions and introduce pregnant women to talk more.

### The final intervention protocol

Table 4 displayed detailed information about the 13 sessions of the final intervention protocol. Opinions from the expert panel were accepted. Combining the experts’ opinions and an RCT [53], the number of pregnant women in groups is identified as 8–10.

**Table 4:** The final intervention protocol

Sessions	Justification	Content	Time	Delivery type	Duration	Intervener
Session 1	knowledge education session	(1) What is GDM? (2) Adverse health outcomes of GDM? (3) Physical activity could prevent GDM. (4) The frequence, intensity,type, time of physical activity during pregnancy (5) Saftey precautions when doing physical activity (6) Healthy pregnancy weight gain	13–14^+ 6^ weeks’ gestation	face to face in groups with 8–10 participants at the antenatal clinic	about 30 min	Researcher, nurse and midwife
Session 2	exercise clinic visit	(1) the coach teach pregnant women how to exercise safely (2) the coach guide the participants to do physical activity together following the exercise video. (3) uploaded the exercise video to WeChat platform (4)teach the participants how to keep exercise diary and record everyday’s weight	13–14^+ 6^ weeks’ gestation	face to face in groups with 8–10 participants at the antenatal clinic	about 60 min	Researcher,coach, obstetricians, nurses, and midwife
Session 3	pregnancy-related symptom management	(1) Assessing and explaining the participant’s pregnancy-related symptoms, (2) strategies for managing symptoms such as muscle relaxation	15–16^+ 6^ weeks’ gestation	face to face in groups with 8–10 participants at the antenatal clinic	about 40 min	Researcher, nurses, and midwife
	group discussion	(1) Checking behavioral tracking, review, and feedback in the prior 2 weeks (2) Sharing self-management strategies from successful pregnant women (3) recalling previous successful experience, problem solving, and encouraging				
Session 4	pregnancy related emotion management	(1)Assessing and explaining the participant’s pregnancy-related negative emotions (2)strategies for managing anxiety, or depression, such as positive self-talk	17–18^+ 6^ weeks’ gestation	online group discussion with 15–20 participants via Tencent Meeting	about 40 min	Researcher, nurses, midwife, and psychological consultant
	group discussion	(1) Checking behavioral tracking, review, and feedback in the prior 2 weeks (2) Sharing self-management strategies from successful pregnant women (3) recalling previous successful experience, problem solving, and encouraging				
Session 5–13	group discussion	(1) positive feedback on gestational weight gain and physical activity in the prior 2 weeks (2) role model: sharing self-management strategies from successful pregnant women (3) problem solving, discuss the problems that arise when doing exercise and share solutions on how to keep active with each other (4) recall previous successful experience, guiding participants to recall previous successful behavior-change situations, discuss context and factors associated with success (5) Encourage:confirming participants have the capability for exercise and weight self-management	every two weeks from 19–20^+ 6^ to 37 weeks	face to face discussion at the antenatal clinic or online discussion via Tencent Meeting in groups with 8–10 participants	about 30 min	Researcher, nurses, and midwife

Note: GDM, gestational diabetes mellitus

Previous studies indicated that interventions delivered face-to-face were significantly associated with larger effect sizes in both post-intervention changes and maintained changes in physical activity self-efficacy [75]. Combining the experts’ opinions on delivery type, more face-to-face session was added. The session on pregnancy-related symptom management will be conducted face-to-face followed by a group discussion. The session on pregnancy-related emotion management will be conducted online via Tencent Meeting followed by group discussion. The rest of the follow-up group discussion sessions will be conducted face-to-face at the antenatal clinic or online via Tencent Meeting.

Safety precautions including exercises to avoid during pregnancy, safety considerations, and warning signs to discontinue exercise during pregnancy were added to the first session of knowledge education. Furthermore, safety concerns will be emphasized in each intervention session. In addition, information about healthy pregnancy weight gain was provided in the first session of knowledge education. The participants were asked to record every day’s weight in the exercise diary.

During the group discussion sessions, the intervener will be responsible for analyzing the participants’ weight gain and physical activity in the prior 2 weeks. Then 2 pregnant women will be invited as role models to share their successful self-management strategies to achieve physical activity goals. To make more pregnant women stand out to be models, the 2 role models are different in each group discussion session. Then the intervener will guide participants to discuss the problems that arise when doing exercise, share solutions on how to keep active with each other, recall the previous successful experience, and discuss the context and factors associated with success. Finally, the intervener will encourage participants to confirm their capability for exercise and weight self-management.

### The feasibility and acceptability of the intervention

A single-blinded randomized controlled trial (RCT) was conducted. Subsequently, 34 pregnant women with high risk for GDM were randomized for the intervention (n = 17) or the control group (n = 17).

#### Characteristics of the pilot sample

The characteristics of the participants are summarised in Table 5. The mean age of the entire sample was 33.59 (SD = 4.19). The mean gestational age of the entire sample was 12.56 (SD = 0.79). 82.4% received a university degree or above, 47.6% were overweight or obese with pre-pregnancy BMI above 24 Kg/m^2^, 64.7% were multiparas, 85.3% had a planned pregnancy, and 61.7% exercised regularly before pregnancy. No significant difference in baseline characteristics was found between the intervention and control groups.

**Table 5:** Comparison of demographic characteristics and outcome variables between intervention (n = 17) and control groups (n = 17)

Variables	Overall	Intervention group	Control group	*t*/χ^2^	*P*
Age (years) (range: 25–41)	33.59 ± 4.19	34.00 ± 4.17	33.18 ± 4.31	0.567	0.575
<35	16	7	9	0.472	0.492
≥ 35	18	10	8		
Gestational age (weeks) (range: 11–14)	12.56 ± 0.79	12.47 ± 0.80	12.65 ± 0.79	-0.649	0.521
Gestational weight gain (Kg) (range: -3-7.9)	2.00 ± 2.42	2.32 ± 2.39	1.67 ± 1.87	0.768	0.448
Pre-pregnancy BMI (Kg/m^2^)				3.256	0.354
<18.5	2	1	1		
18.5–23.9	16	10	6		
24-27.9	13	4	9		
≥ 28	3	2	1		
**Education**				0.000	1.000
High school or below	6	3	3		
University degree or above	28	14	14		
**Employment**				4.963	0.084
Housewife	7	1	6		
Part time	4	3	1		
Full time	23	13	10		
**Monthly household income** (per person per month)				0.917	0.632
<¥5000 (about US$747)	12	6	6		
¥5000–9000 (about US$747–US$1344)	16	7	9		
≥¥9000 (aboutUS$1344)	6	4	2		
**Number of pregnancies**				3.767	0.439
1	5	3	2		
2	15	8	7		
3	8	3	5		
4	4	3	1		
5	2	0	2		
**Parity**				2.866	0.239
First delivery	11	7	4		
Second delivery	21	10	11		
Third delivery	2	0	2		
**Family history of type 2 diabetes**				3.238	0.072
Yes	6	5	1		
No	28	12	16		
**Polycystic ovary syndrome**				1.030	0.310
Yes	1	1	0		
No	33	16	17		
**Previous macrosomia**				0.000	1.000
Yes	2	1	1		
No	32	16	16		
**Previous fetal anomaly**				0.134	0.714
Yes	11	5	6		
No	23	12	11		
**Previous hydramnion**				1.030	0.310
Yes	1	1	0		
No	33	16	17		
**Repeated colitis**				0.000	1.000
Yes	2	1	1		
No	32	16	16		
**Planned pregnancy**				0.234	0.628
Yes	29	15	14		
No	5	2	3		
**Having a habit of regular physical activity before pregnancy**				1.121	0.290
Yes	11	5	6		
No	23	12	11		
**Learning physical activity knowledge**				1.074	0.300
Yes	15	7	8		
No	19	10	9		
**The incidence of GDM**	32.3% (10/31)	26.7%(4/15)	36.5% (6/16)	0.416	0.519

#### Feasibility of the intervention

The participants were recruited from the antenatal clinic of the study hospital in Zhengzhou. Once pregnant, a medical record will be established after a comprehensive assessment. A total of 217 pregnant women were screened for eligibility from February to March 2022. Forty-one of them met the inclusion and exclusion criteria. Finally, 34 of the 41 eligible pregnant women consented to participate in the study. Thus, an 82.9% recruitment rate was achieved in this pilot study.

**Fig. 2:**
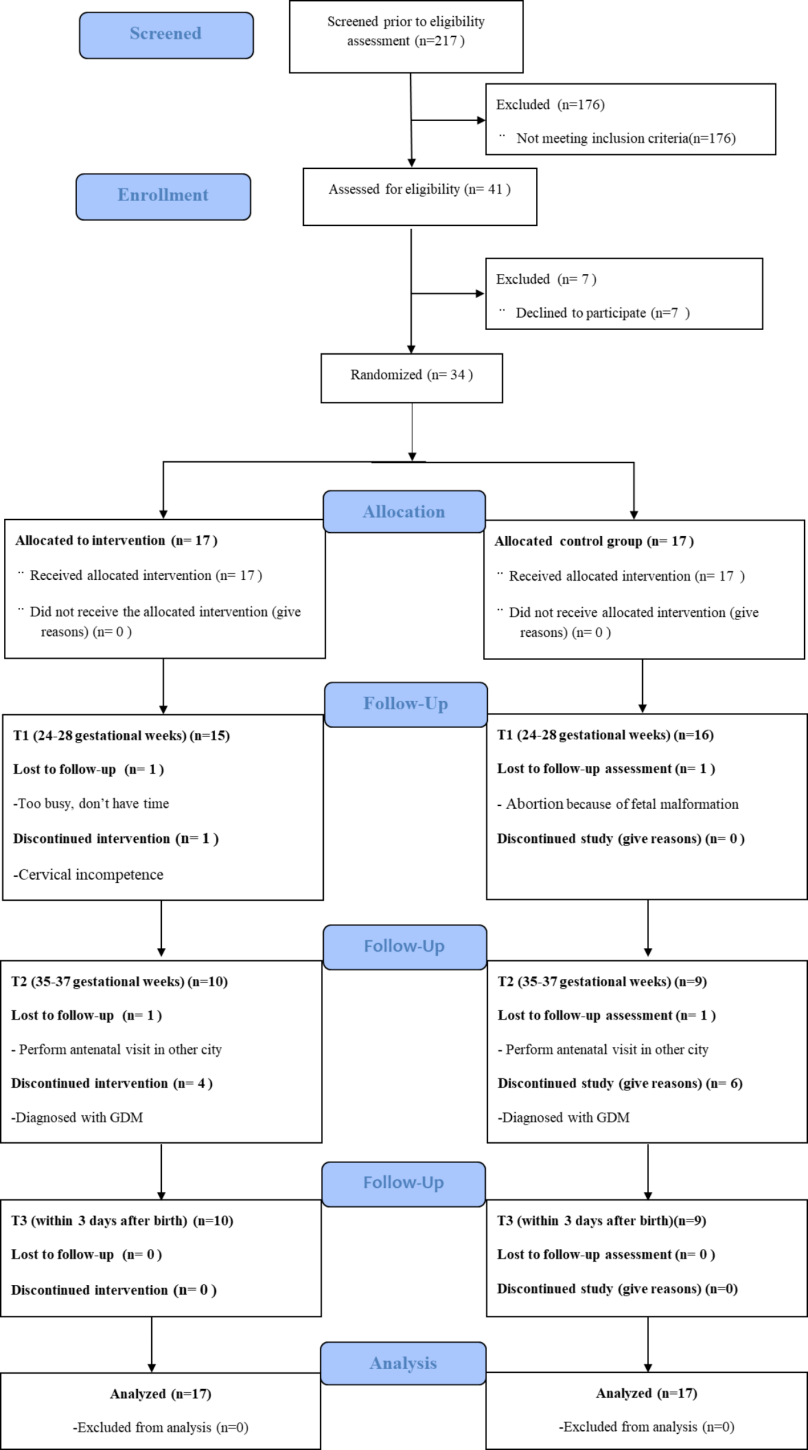
Flow diagram of participant recruitment

As shown in Figs. 2 and 31 (31/34, 91.2%) pregnant women complete the follow-up questionnaires in the first follow-up during 24–28 gestational weeks. A total of 10 pregnant women were diagnosed with GDM and then followed the treatment measure of GDM. The incidence of GDM in the intervention group (26.7%, 4/15) was lower than that in the control group (37.5%, 6/16) (*t* = 0.416, *p*>0.05). The 10 pregnant women with GDM discontinued the study. Finally, 19 (19/34, 58.9%) pregnant women completed the follow-up questionnaires in the second follow-up during 35–37 gestational weeks and the third follow-up within 3 days after birth.

Concerning the participation in the intervention protocol, all the retention pregnant women had completed 11 to 13 intervention sessions. Concerning the completion rate of the intervention protocol, all the retented participants completed 80% of the total intervention sessions.

#### Acceptability of the intervention

The participants’ responses to the 8-item satisfaction questionnaire are presented in Table 6. All the participants are satisfied with the intervention protocol.

**Table 6:** Participants’ responses regarding their satisfaction with the intervention (N = 15)

	range	Mean ± SD	Agree or strongly agree (%)
1. This program is implementable.	3–5	4.67 ± 0.49	100
2. This program is easy and clear to understand.	3–5	4.80 ± 0.41	100
3. I welcome this program	3–5	4.73 ± 0.46	100
4. This program is effective.	3–5	4.80 ± 0.41	100
5. This program meets my needs.	3–5	4.73 ± 0.46	100
6. I am satisfied with this program.	3–5	4.67 ± 0.49	100
7. I like to continue this program.	3–5	4.73 ± 0.46	100
8. This program is good to extend.	3–5	4.86 ± 0.35	100

## Discussion

According to the MRC framework, this study successfully developed and pilot-tested a theory-guided and evidence-based physical activity intervention in women with high risk for GDM. The intervention development process is comprehensively supported by theory, clinical practice recommendations, evidence from systematic reviews and RCTs, consideration of context’s characteristics and economics, and healthcare providers’ multi-professional involvement as key stakeholders to identify the content validity of the intervention protocol. The intervention development process ensures the intervention protocol is research-informed, theoretically appropriate, and practically feasible. The pilot clinical trial further indicated the feasibility and the acceptability of the intervention protocol.

Systematic reviews of clinical practice guidelines [14] and RCTs [48–59] were conducted by the research team for the identification of the essential elements of physical activity training. The elements of physical activity training also took into consideration the participants’ characteristics in Chinese traditional culture and antenatal visit schedules in mainland China. Finally, pregnant women with high risk for GDM were asked to perform regular physical activity 30 min per session for at least 5 days in moderate intensity from 13–14^+ 6^ gestational weeks to 37 gestational weeks with warm up and cool down. The participants could begin to perform physical activity for 10 min, and then gradually increase to 30 min. The data will be collected at four time points: baseline (T0), 24–28 gestational weeks (T1), 35–37 gestational weeks (T2), and 3 days after delivery (T3).

This physical activity intervention protocol for pregnant women with GDM is developed based on the self-efficacy theory [42]. Besides the four sources of self-efficacy, this study combined effective BCTs on physical activity self-efficacy addressed from five systematic reviews [75, 77–80] and a review of BCTs on physical activity during pregnancy [81]. Therefore, the strategies to improve self-efficacy in the present study were comprehensively applied based on theory and evidence.

The intervention protocol was developed as blended interventions together with face-to-face and mHealth. Based on evidence and the communication habits of the Chinese population, WeChat and Tencent meetings were selected as delivery channels for daily communication and online group discussion sessions, respectively. In addition, the physical activity video will be uploaded on the small program of WeChat. Participants could follow the video to perform physical activity every day. Then they could record their daily weight and exercise diary on the small program of WeChat. The blended interventions in the present study would be more convenient and cost-effective for participants.

In addition, excellent reliability and content validity of the intervention protocol was demonstrated among the expert panel. To promote physical activity during pregnancy, obstetric care providers and exercise specialists are recommended to cooperate closely [84]. In this study, we selected experts from medicine, nursing, midwives, sports, physiotherapy, and psychology to form a multidisciplinary team. Their rich teaching and clinical experience ensured an in-depth understanding of the related themes. The high authority coefficient of the experts in the present study indicated that the experts are very familiar with the research topic and have high authority. The expert consultation revealed significant coordination coefficients, indicating the experts’s high coordination degree concerning all the indicators. The content validity assessment of the physical activity intervention protocol demonstrated an excellent outcome. All items reached satisfactory scores in the one round of assessment. Following the experts’ suggestions and further evidence, a final thirteen-session physical activity intervention was created.

To improve the intervention and study design, a pilot trial was designed to examine the feasibility and the acceptability of the intervention protocol. The utilization of the intervention protocol was found to be feasible, with a high recruitment rate of the eligible participants, retention rate of the enrolled participants, and completion rate of the total intervention sessions. The lower incidence of GDM in the intervention group highlights the necessity to improve physical activity to prevent GDM in pregnant women with a high risk for GDM. There was one participant dropped from the study due to the contraindications of exercise during pregnancy. This information reminds researchers to dynamically evaluate pregnancy progress and closely consider the medical records of the participants. The participants should know safety precautions regarding physical activity during pregnancy.

Concerning acceptability, the respondents to the satisfaction questionnaire were great. The blended intervention was described as easy and clear to use, and easy to understand. The participants claimed that this program met their needs and was helpful. They were willing to recommend this program to other pregnant women. High satisfaction rates (100%) were achieved in this intervention.

### Clinical implications

To the best of our knowledge, this is the first pilot trial study that incorporates self-efficacy theory, enhancing strategies, and blended methods to promote physical activity in pregnant women with high risk for GDM. Physical activity can prevent the incidence of GDM and improves health outcomes for pregnant women and their offsprings. This theory-guided, evidence-based, and blended physical activity intervention appeals to be delivered to a large number of participants. Additionally, this intervention is developed step by step under the guidance of the MRC framework. The present study provides an example of a process for intervention protocol development and pilot testing, and can also be applied as a learning reference for clinical research. Apart from that, this study may improve the cooperation of medicine, nursing, and sports. The study results will help to build normal procedures for the management of physical activity during pregnancy.

This study has some limitations. The expert panel discussion was held online, which may cover some nonverbal communication [85]. To increase the visualization of the covered information uncovered, measures were taken before and during the meeting. Before the meeting, the materials were sent to the experts for their familiarity with the study content. During the meeting, an experienced host guided the experts to share their opinions as much as possible. The acceptability of the intervention protocol was collected by a numbered questionnaire in the study. It is suggested to ask open-ended questions to determine the acceptability outcomes in the following large RCT. The non-satistical difference of GDM rates between the two groups may be due to the small sample size in this pilot study. Additionally, as the participants in the pilot study were with a high level of education and good socioeconomic conditions, they would likely be motivated to comply with the protocol recommendations. Participants with low education levels should be involved in further study. The data analysis should be conducted through hierarchical analysis with a large sample size in the following large RCT.

## Conclusion

The present study successfully developed a theory-guided and evidence-based physical activity intervention in women with high risk for GDM following the MRC framework. The study results indicate that the developed self-efficacy-enhancing physical activity intervention is both clinically feasible and acceptable to be used by pregnant women with high risk for GDM. Following this pilot study, we are planning to conduct a large RCT to compare the effectiveness of the final version of the intervention with the usual prenatal care in pregnant women at high risk for GDM. Findings from the large RCT are expected to provide the foundation for health policymakers and healthcare providers to shape standardized physical activity monitoring procedures in antenatal care, and then increase physical activity during pregnancy and decrease the burden of non-communicable diseases.

### Electronic supplementary material

Below is the link to the electronic supplementary material.

Supplementary Material 1

Supplementary Material 2

## Data Availability

All data generated or analyzed during this study are included in this published article and its supplementary information files.

## Abbreviations

BMI: Body mass index
EDC: Expected date of confinement
GDM: Gestational diabetes mellitus
mHealth: Mobile health
RCT: Randomized controlled trial
