# Supplementary material for: Development and feasibility of a theory-guided and evidence-based physical activity intervention in pregnant women with high risk for gestational diabetes mellitus: a pilot clinical trial
Source: BMC Pregnancy Childbirth. 2023 Sep 19;23:678. doi: 10.1186/s12884-023-05995-7 (PMC10510212; doi:10.1186/s12884-023-05995-7)
Supplement: Supplementary file 1 — Supplementary Material 1 [file 12884_2023_5995_MOESM1_ESM.docx]

**Screenin**

**g**

**Included**

**Eligibilityg**

**Identification**

Guidelines met eligibility criteria (n =12)

Full-text articles assessed

for eligibility (n =64)

Records screened

(n =64)

Records after duplicates removed

(n =1196 )

Additional records identified

through other sources

(n =6)

Records identified through websites and database searching (n = 1950)

Duplicates removed

(n =760)

Records excluded based

on title and abstract

(n =1032)

Full-text articles excluded

(n =52)

No detailed intervention strategies

Already had the English version

Pilot study

Figure S1 Literature screening flow chart
