# Supplementary material for: Development and feasibility of a theory-guided and evidence-based physical activity intervention in pregnant women with high risk for gestational diabetes mellitus: a pilot clinical trial
Source: BMC Pregnancy Childbirth. 2023 Sep 19;23:678. doi: 10.1186/s12884-023-05995-7 (PMC10510212; doi:10.1186/s12884-023-05995-7)
Supplement: Supplementary file 2 — Supplementary Material 2 [file 12884_2023_5995_MOESM2_ESM.docx]

Table S1 Risk of Bias Summary

| Study | The Cochrane tool for bias risk assessment | | | | | | | |
| --- | --- | --- | --- | --- | --- | --- | --- | --- |
|  | random sequence generation | allocation  concealment | baseline outcomes | blinding of participants and personnel | blinding of outcome assessment | incomplete outcome data | selective reporting | Other sources of bias |
| Bisson et al., 2015 | low risk | low risk | low risk | high risk | low risk | low risk | low risk | unclear risk |
| Barakat et al., 2013 | low risk | low risk | low risk | high risk | low risk | low risk | low risk | unclear risk |
| Wang et al., 2017 | low risk | low risk | low risk | high risk | unclear risk | low risk | low risk | unclear risk |
| Seneviratne et al., 2016 | low risk | low risk | low risk | high risk | unclear risk | low risk | low risk | unclear risk |
| Guelfi et al., 2016 | low risk | unclear risk | low risk | unclear risk | unclear risk | low risk | low risk | unclear risk |
| Ruiz et al., 2013 | low risk | unclear risk | low risk | unclear risk | unclear risk | low risk | low risk | unclear risk |
| Garnæs et al., 2016 | low risk | low risk | low risk | low risk | unclear risk | low risk | low risk | unclear risk |
| Kong et al., 2014 | low risk | low risk | low risk | high risk | unclear risk | low risk | low risk | unclear risk |
| Daly et al., 2017 | low risk | low risk | low risk | low risk | unclear risk | low risk | low risk | unclear risk |
| Oostdam et al., 2012 | low risk | low risk | low risk | low risk | low risk | low risk | low risk | unclear risk |
| Simmons et al., 2017 | low risk | low risk | low risk | low risk | unclear risk | low risk | low risk | unclear risk |
| Callaway et al., 2010 | unclear risk | high risk | low risk | high risk | unclear risk | low risk | low risk | unclear risk |

Note: Each item was assessed as (1) high risk, (2) low risk and (3) unclear risk by applying the Cochrane criteria for judging bias risk. The risk of bias of each study was assessed as (1) high risk if one or more key domains were low risk; (2) low risk, if all key domains were low risk or (3) unclear risk, if one or more key domains were unclear.

Table S2 Characteristics of the Expert Panel Members

| Characteristics of the Panel Experts (n = 10) | Number of Experts | Percentage (%) |
| --- | --- | --- |
| **Gender** |  |  |
| Male | 2 | 20.0 |
| Female | 8 | 80.0 |
| **Age** |  |  |
| 30 to 40 years old | 4 | 40.0 |
| 40 to 50 years old | 5 | 50.0 |
| 50 to 60 years old | 1 | 10.0 |
| **Profession** |  |  |
| Medicine | 1 | 10.0 |
| Nursing | 4 | 40.0 |
| Midwifery | 1 | 10.0 |
| Physiotherapy | 1 | 10.0 |
| Sports | 2 | 20.0 |
| Psychology | 1 | 10.0 |
| **Institution** |  |  |
| University | 3 | 30.0 |
| Hospital | 7 | 70.0 |
| **Academic professional rank** |  |  |
| Full professor, chief physician, and chief nurse | 4 | 40.0 |
| Associate professor and associate chief nurse | 2 | 20.0 |
| Nurse-in-charge and lecturer | 4 | 40.0 |
| **Highest academic qualification** |  |  |
| Doctorate degree | 1 | 10.0 |
| Master’s degree | 6 | 60.0 |
| Bachelor’s degree | 3 | 30.0 |
| **Years of professional experience** |  |  |
| More than 20 years | 5 | 50.0 |
| 11 to 20 years | 5 | 50.0 |

Table S3 Authority coefficient of the Expert Panel

| Number | Judgment criteria | | | | Ca | Cs | Cr |
| --- | --- | --- | --- | --- | --- | --- | --- |
|  | Theoretical analysis | Literature reference | Practical experience | Subjective judgment |  |  |  |
| 1 | 0.45 | 0.2 | 0.15 | 0.05 | 0.85 | 0.60 | 0.73 |
| 2 | 0.45 | 0.3 | 0.2 | 0.05 | 1.00 | 0.80 | 0.90 |
| 3 | 0.35 | 0.3 | 0.15 | 0.05 | 0.85 | 0.80 | 0.83 |
| 4 | 0.45 | 0.3 | 0.2 | 0.05 | 1.00 | 1.00 | 1.00 |
| 5 | 0.45 | 0.2 | 0.15 | 0.05 | 0.85 | 0.80 | 0.83 |
| 6 | 0.45 | 0.3 | 0.15 | 0.05 | 0.95 | 0.80 | 0.88 |
| 7 | 0.45 | 0.3 | 0.2 | 0.05 | 1.00 | 1.00 | 1.00 |
| 8 | 0.35 | 0.3 | 0.2 | 0.05 | 0.9 | 1.00 | 0.95 |
| 9 | 0.45 | 0.2 | 0.15 | 0.05 | 0.85 | 0.80 | 0.83 |
| 10 | 0.45 | 0.3 | 0.15 | 0.05 | 0.90 | 0.80 | 0.85 |
| The average Cr = (0.73+0.90+0.83+1.00+0.83+0.88+1.00+0.95+0.83+0.85)/10 | | | | | | | 0.88 |

Note: Cs, familiarity coefficient; Ca, judgment coefficient; Cr, authority coefficient.

Cr = (Cs + Ca)/2.

Table S4 The results of the degree of coordination among experts Kendall’s concordance coefficients

|  | Items(n) | Kendall's W | *χ*^2^ | *p* |
| --- | --- | --- | --- | --- |
| Primary indicators | 4 | 0.400 | 12.000 | 0.007 |
| Secondary indicators | 10 | 0.305 | 27.432 | 0.001 |
| Tertiary indicators | 17 | 0.354 | 56.572 | 0.000 |
| Total | 31 | 0.321 | 96.163 | 0.000 |

Table S5 The results of content validation from the expert panel of the preliminary intervention protocol

| Items | ^a^Mean ± SD | ^b^CV | ^c^CLA (%) |
| --- | --- | --- | --- |
| 1. Accomplishment experiences | 5.00 ± 0.00 | 0.00 | 100 |
| 1.1 knowledge education session | 4.80 ± 0.42 | 0.09 | 100 |
| 1.1.1 What is GDM? | 5.00 ± 0.00 | 0.00 | 100 |
| 1.1.2 Adverse health outcomes of GDM? | 5.00 ± 0.00 | 0.00 | 100 |
| 1.1.3 Physical activity could prevent GDM. | 5.00 ± 0.00 | 0.00 | 100 |
| 1.1.4 The frequence, intensity,type, time of physical activity during pregnancy | 5.00 ± 0.00 | 0.00 | 100 |
| 1.2 exercise clinic visit | 5.00 ± 0.00 | 0.00 | 100 |
| 1.2.1 the coach teach pregnant women how to exercise safely | 5.00 ± 0.00 | 0.00 | 100 |
| 1.2.2 the coach guide the participants to do physical activity together following the exercise video. | 5.00 ± 0.00 | 0.00 | 100 |
| 1.2.3 uploaded the exercise video to WeChat platform | 4.30 ± 0.48 | 0.11 | 100 |
| 1.2.4 teach the participants how to keep exercise diary | 4.20 ± 0.79 | 0.19 | 80 |
| 2. Vicarious experience | 5.00 ± 0.00 | 0.00 | 100 |
| 2.1 positive feedback | 4.70 ± 0.48 | 0.10 | 100 |
| 2.1.1 Checking behavioral tracking, review, and feedback on goals | 4.30 ± 0.48 | 0.11 | 100 |
| 2.2 role model | 4.70 ± 0.48 | 0.10 | 100 |
| 2.2.1 Sharing self-management strategies from successful pregnant women | 4.60 ± 0.84 | 0.18 | 90 |
| 3.Verbal persuasion | 5.00 ± 0.00 | 0.00 | 100 |
| 3.1 reminder | 4.70 ± 0.48 | 0.10 | 100 |
| 3.1.1 Daily reminders via the WeChat group to encourage the women to follow the 40-min video, performing the exercises and recording in their exercise diary | 4.50 ± 0.85 | 0.19 | 80 |
| 3.2 problem solving | 4.80 ± 0.42 | 0.09 | 100 |
| 3.2.1 Discuss the problems that arise when doing exercise and share solutions on how to keep active with each other | 4.70 ± 0.48 | 0.10 | 100 |
| 3.3 encourage | 4.80 ± 0.42 | 0.09 | 100 |
| 3.3.1 Confrming participants have the capability for exercise and weight self-management | 5.00 ± 0.00 | 0.00 | 100 |
| 3.4 recall previous successful experience | 4.40 ± 0.52 | 0.12 | 100 |
| 3.4.1 Guiding participants to recall previous successful behavior-change situations, discuss context and factors associated with success | 5.00 ± 0.00 | 0.00 | 100 |
| 3.5 positive feedback | 5.00 ± 0.00 | 0.00 | 100 |
| 3.5.1 Providing positive feedback for the participant’s effort | 4.20 ± 0.52 | 0.11 | 100 |
| 4. physiological and emotional status | 4.80 ± 0.42 | 0.09 | 100 |
| 4.1 knowledge education session | 4.40 ± 0.52 | 0.12 | 100 |
| 4.1.1 Assessing and explaining the participant’s pregnancy-related symptoms, and discussing strategies for managing symptoms, such as muscle relaxation | 4.70 ± 0.48 | 0.10 | 100 |
| 4.1.2 Assessing and explaining the participant’s negative emotions, and discussing strategies for managing anxiety, or depression, such as positive self-talk | 4.70 ± 0.48 | 0.10 | 100 |

Note: ^a^ SD: standard deviation. ^b^ CV: coefficient of variation. ^c^ CLA: the percentage of participants who rated an item as 4 or 5 points.
